# Major Changes in a Rhythmic Ball-Bouncing Task Occur at Age 7 Years

**DOI:** 10.1371/journal.pone.0074127

**Published:** 2013-10-02

**Authors:** Christophe Bazile, Isabelle A. Siegler, Nicolas Benguigui

**Affiliations:** 1 Laboratoire CIAMS (Complexité, Innovation, Activités Motrices et Sportives), Univ Paris-Sud, Orsay, France; 2 Normandie University, Caen, France; 3 Laboratoire CESAMS (Centre d'Etude Sport et Actions MotriceS), UNICAEN, Caen, France; University of Reading, United Kingdom

## Abstract

The aim of the study was to investigate the development of a rhythmical skill of children aged from 5 to 12 years old. Five age groups (5–6, 7–8, 9–10, 11–12, and young adults) performed a virtual ball bouncing task (16 forty-second long test trials). Task performances, racket oscillation, ball-racket impacts as well as the ball-racket coupling were analysed. The results showed a change in both performance and behaviour at the age of 7 years old. Before this age, children exhibited restricted perceptual-motor coordination with a high frequency of racket oscillation and a poor level of performance. After the age of 7, cycle-to-cycle adaptive coordination based on visual information was progressively acquired leading to increasing performance levels with age. Overall these results revealed a rapid change in capability to perform the ball bouncing task across age with a late emergence of the required coordination and significant change in the coordination at the age of 7.

## Introduction

A fundamental characteristic of human behavior is the ability to attune movement to environmental constraints [1]. The capability to control goal-oriented movements within these constraints is a crucial challenge for sensorimotor development [2], [3]. The movements exhibited by infants, primarily waving, kicking or sucking, are spontaneous self-paced coordination patterns called stereotypies [4], [5] and progress with age toward the adaptive capability to control rhythmic motor coordination on the basis of perceptual information provided by the environment [6].

The main goal of the present study is to investigate the development of rhythmic visual-motor coordination in primary school-aged children which has never been studied until now. A ball-bouncing task was chosen since it requires the production of a rhythmic arm motion synchronized to the ball. Despite the absence of related literature, research informative of the various elements of ball bouncing exists on similar perceptual-motor tasks, including catching and tapping. Infants' ability to catch a moving object starts very early in life with the simultaneous use of the two hands [7]–[9]. Improvements are seen with age [10], [11], with critical changes between 5 and 12 years old. Indeed, by the age of 5, the one-handed catching skill starts to emerge [12], although Alderson, Sully, & Sully noted that one-handed catching still appears difficult for 7-year old children who spontaneously prefer to catch with two hands [13]. From the age of 8 years old, one-handed catching skill progressively improves until at age 12 being comparable to adult level [11]–[13].

Studies on children's finger tapping address the development of rhythmical acoustical-motor coordination and synchronization to a stimulus. Smoll showed that children at the age of 5 are weak at synchronizing finger tapping to a periodical auditory stimulus, but become increasingly able to do so between the age of 5 and 11 [14]–[16]. Volman & Geuze examined age-related differences in the stability of uni-manual rhythmic patterns [6]. Children aged 7, 9 and 11 years old were asked to synchronize finger tapping to the beat of an auditory metronome either “on the beat”, or “off the beat”. Results showed an age-related increase in learning speed as well as an improvement of both accuracy and stability of the perceptual-motor pattern. These results are widely shared by literature with previous evidence in drawing [17], pointing [18], reaching [19] or intercepting [20] showing that substantial changes in perceptual-motor development in complex skills occur mainly between 5 and 12 years old.

Another constant feature observed in the literature related to the development of perceptual-motor coordination is the occurrence of significant changes during a short period of time at the age of 7–8. In a set of pointing tasks, a nearly total absence of visually-based regulation of movement was shown in children aged from 5 to 7 years old. Then, from 7 to 12 years old, visually-guided regulation of the movement progressively increases with age [19]. It was also shown that stability of motor performance significantly improves after the age of 7 [21]–[23] as does accuracy [18], [20], [23], [24]. Significant improvements in perception and reaction time have also been observed after the age of 7–8 years old [20], [25], [26]. In sum, these results confirm that the age of 7–8 years old can be considered as a sensitive period for development of perceptual-motor coordination with significant qualitative and quantitative changes.

In this context, we used a virtual ball-bouncing task in order to explore the development of rhythmic visual-motor coordination. This task, which consists in rhythmically hitting a ball in a virtual environment, was previously used in order to study the role of visual information in the control of racket motion in adults [27], [28]. Participants are instructed to manipulate a physical table tennis racket that controls a virtual racket so as to get the ball as close as possible to a target placed at a constant height. The ball-bouncing task thus involves the dynamics of goal-oriented behaviour as well as the regulation of racket movement based on sensory information, referred to as the information-movement coupling [29]. Indeed, rhythmically hitting a ball to a constant height implies adequately controlling the movement for the force applied to the ball upon impact to be in accordance with its kinematic properties. A virtual environment was used in the present experiment in order to limit the ball and racket trajectories to a single vertical dimension and to prevent the ball from falling off the racket to the ground. This was expected to make this bouncing task doable for young children. Such set-ups were used in previous studies in adults in which the loss of haptic information on ball-racket impacts was not found to impair the ability to control ball bouncing [27], [28], [30], [31].

The development of sensorimotor synchronization to visual stimulus, such as a ball motion, is maybe different from that of acoustical-motor synchronization, since a difference between the two sensory modalities remains in adulthood. Indeed, it has long been shown that it is more difficult for adults to synchronize with a sequence of flashes than with a sequence of tones, and the auditory modality was shown to be dominant in the temporal processes involved in sensorimotor synchronization tasks (see [32] for a review). However, it has recently been shown that visual-motor synchronization improves substantially with moving visual stimuli such as a continuously bouncing ball [33]. These recent results in adults justify studying the development in children of visual-motor and rhythmic tasks such as ball bouncing. Potential differences in the developmental dynamics, compared to the development of rhythmical motor coordination and synchronization to an auditory stimulus may be expected.

The present study aimed to investigate the development of a rhythmic visual-motor skill in children aged from 5 to 12 years old. A general trend characterized by an improvement of both accuracy and stability of the perceptual-motor behaviour and an increase of performance are expected across age, with a substantial improvement of these characteristics expected by the age of 7–8 years old [18]–[22], [34]. Following published literature related to other tasks, we hypothesized that children under 7 would show a restricted visual-motor coordination characterized by a relatively “fixed” frequency racket oscillation and a weak performance. After the age of 7 a more accurate performance with a release of racket frequency would testify to a progressive involvement of information-movement coupling in task regulation [18]–[22], [34]. Finally, this innovative experiment could provide insight into the temporal visual information involved in the control of effector period (or frequency) which is crucial for such rhythmic coordination.

## Materials and Methods

### Participants

Fifty participants were divided into five age groups (years of age) of 10 participants: Age 5–6 (6.1±0.4 yrs; 4 males, 6 females); Age 7–8 (7.7±0.2 yrs; 5 males, 5 females); Age 9–10 (9.8±0.3 yrs; 5 males, 5 females); Age 11–12 (12.1±0.4 yrs; 6 males, 4 females); Adults (26.4±6 yrs; 5 males, 5 females). All children were recruited in a recreational centre while adults were students at University. No participants reported regular practice in sports involving racket. No motor or perceptual impairments were reported by participants or their parents. After being informed about the experimental procedure, the children's parents as well as young adults provided written informed consent as required by the Helsinki declaration and the EA 4532 local Ethics Committee of University Paris-Sud who specifically approved this study. Furthermore, children were invited to explicitly express their agreement about their participation prior to testing.

### Virtual reality apparatus and data collection

Participants were placed in an upright standing position in front of a screen (1.70 m ×1.70 m) at a distance of 1.5 m. Participants held a table tennis racket, referred to as the “physical racket”, which could be manipulated without any restriction (see [30] for illustration and details). Participants were asked to keep the physical racket horizontal and to perform movements in the vertical dimension only. A sheet of cardboard was positioned horizontally at neck level in order to prevent participants from seeing the physical racket once the experiment began. The position of the physical racket was measured by an electromagnetic sensor (Flock of Birds (FOB), Model 6DFOB, Ascension technologies) with a sampling rate of 120 Hz. The sensor was fixed on the backside of the physical racket while the transmission base of this device (serving as a space reference) was fixed to a stand so that they directly faced each-other. The signal was sent to custom-written experimental software (see [35] for more details) in the host computer. From the in-line position of the physical racket, the software computed the position of a “virtual racket”, which was materialized by a horizontal bar (0.20 m wide ×0.025 m high) and displayed on the screen using a video projector (Epson EH-TW 450, 3LCD, 50 Hz). The software also computed the position of a “virtual ball” (diameter  = 0.04 m) and its interactions at impact with the racket trajectory. The end-to-end visual latency between the physical and the virtual racket motion was 29.78±1.07 ms (see [35] for more details). Therefore participants were able to control the virtual racket motion by manipulating the physical racket in order to “virtually” hit the ball. A sound was played at ball -racket impact. The two critical environmental parameters affecting the dynamics of the ball-racket system [36], namely the coefficient of restitution (α = 0.50) and gravity (g = 9.81 m/s^2^), remained unchanged throughout the experiment.

### Procedure

Participants were first shown a short demonstration of the ball bouncing task. They were then asked to stand upright facing the screen and to hold the racket with their preferred hand, keeping it still with elbow flexion at about 90° for five seconds. The racket position was recorded and used as the racket zero position. Participants were instructed to rhythmically bounce the virtual ball with the racket for the duration of a trial so that, at its peak, the ball would rise as close as possible to a virtual target presented on the screen as a horizontal line. The target was positioned at a height H = 0.65 m above the racket zero position. Each trial lasted 40 s and participants had to perform 16 trials during the experiment. A short break at midsession was programmed and participants were free to ask for additional breaks. The experiment lasted approximately 25 min.

### Data reduction and analyses

Raw racket position data were filtered using a second-order Butterworth filter with a cut-off frequency of 12 Hz. Filtered racket position values were symmetrically differentiated to yield racket velocity and again differentiated to obtain racket acceleration. The first 4 s of each trial were systematically discarded from the analysis. For each trial, dependent variables were computed with a custom-written Matlab (Mathworks) program in order to analyze both task performance and behaviour.

**Figure 1:**
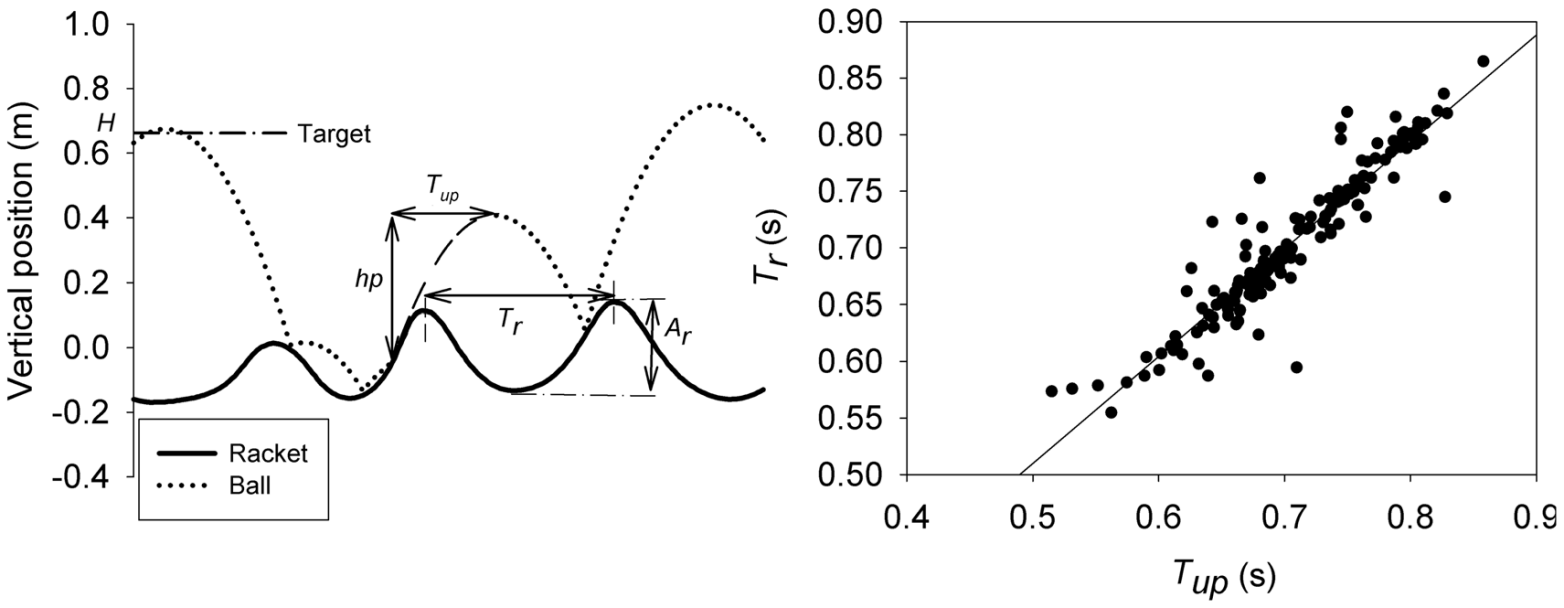
Methods. Left. Definition of variables for racket motion (solid line) and ball trajectory (dotted line). *T_r_* and *A_r_* refer to racket period and amplitude of racket cycle, respectively. *T_up_* and *h_p_* refer to the ball upswing duration and the ball peak height, respectively; Right. Illustration of linear regression between *T_r_* and *T_up_* allowing to determine a coefficient of correlation *R_c_* between both variables.

Mean and standard deviation of ball peak height (*h_p_, SDh_p_*) characterized performance since the task for the participants was to rhythmically hit the ball so that the peak of its trajectory came as close as possible to the visual target (*H* = 0.65 m). Accuracy and stability in performance were computed since they are both known to increase with age when children learn a perceptual-motor skill [17], [23], [24]. Moreover, participants were expected to perform the ball-bouncing task by hitting the ball only once per racket cycle, whose length is defined by two consecutive maxima of racket position (e.g avoid multiple impacts in a racket cycle, or racket cycles void of impacts). Indeed, this specific 1:1 ball-racket coordination is naturally performed by adults who exploit the physics of the system to enact a stable solution [29], [31], [36]. This is the reason why ball-racket impacts needed to be carefully analysed in order to track chaotic bouncing with multiple impacts per racket cycle (for an example see Figure 1 left). The dependent variables characterizing ball-racket impacts within a trial were the mean number of impacts per racket cycle (*Mean_Imp*), the percentage of racket cycles during which no impact (*Imp0*), one impact (*Imp1*) or more than one impact (*Imp+*) occurred.

In order to investigate age-related increase of periodic behaviour stability (e.g. [21], [34]), racket oscillations were characterized for each trial by the mean and standard deviation of racket frequency (*f_r_*, *SDf_r_*) and mean amplitude (*A_r_*). Racket frequency was computed as the inverse of cycle period *T_r_*, defined as the time between two consecutive maxima of racket position [31].

The racket amplitude of a cycle was defined as the difference between the minimum and the first following maximum of the racket position within a racket cycle (upswing amplitude). In order to gain better insight into the “shape” and stability or variability of racket cycles as a function of Group and trial repetition, racket velocity profiles and the standard deviation of velocity profiles were computed. To do so, the continuous time series of racket velocity were segmented into cycles. All the cycles of a trial were time-normalized, interpolated and divided into 20 bins. For each bin, average and the standard deviation across trial were determined. Then, for each group, the mean velocity profile and mean variability of velocity profiles were computed for Trials 1 to 16.

Following Siegler et al. [27], [28], who characterized the coupling between visual information and racket movement in adult ball bouncing, we computed the correlation *R_c_* between the duration of ball motion following the last impact of each cycle (of a trial) *T_up_* and the period *T_r_* of the cycle following this impact (Figure 1, Left). The *R* values of the 16 trials were converted into Z values using Fischer's Z transformation in order to enable averaging and statistical analyses. In order to plot figures, the group means of Z values were converted back into “mean” R values with the inverse of the Fischer's Z transformation (Figure 1, Right). An age-related increase of *Rc* is expected, due to the emergence of a progressive information-movement coupling in children as shown by literature (e.g. [14]–[18], [34]).

### Statistical analyses (Statsoft Statistica 6.0 software)

Two-way mixed-model ANOVAs (5 age groups ×16 trials) with repeated measures on Trial factor and age group as a between factor were used to investigate the effects on dependent variables characterizing performance, ball-racket impacts, racket oscillation and information-movement coupling. In order to determine if racket frequency significantly differed from the gravity induced frequency, *t* tests were performed between the mean racket frequency per trial and the reference value of 1.37 Hz (which is the frequency of a ball bouncing vertically under the effect of gravity with an impact occurring at the height of 0 m and a peak height of 0.65 m). The alpha level was set at p = .05. Post-hoc Tukey's HSD tests were used when needed. For all the post-hoc mean comparisons reported in the result section, the size effect (Cohen's d) was computed and compared to the .80 threshold, which is considered to show a large size effect [37].

## Results

### Task performance

**Figure 2:**
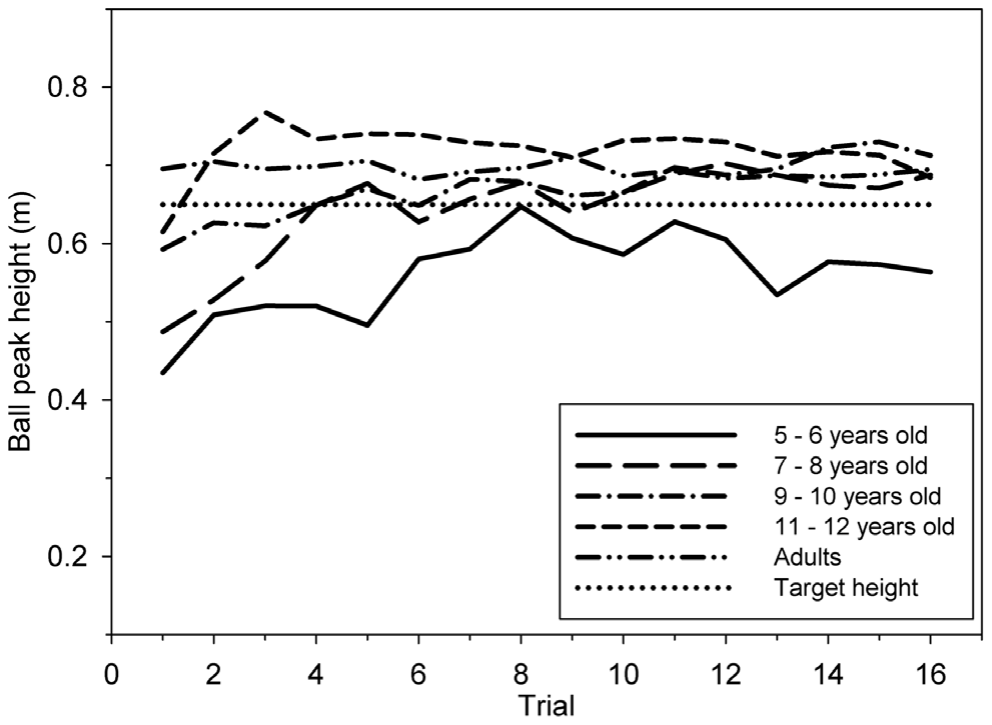
Mean ball peak height *h_p_* throughout 16 trials for the 5 age groups.

Figure 2 displays how mean ball peak height evolved as a function of trial repetition across the five groups. The youngest children (Age 5–6) bounced the ball to a lower height than the other groups: *h_p_* increased from Trial 1 to Trial 6, and then reached a performance plateau. Age 7–8 also exhibited an initial increase of performance during the very first trials, whilst the remaining age groups exhibited stable performance from the beginning of the experiment. A mixed model analysis of variance (ANOVA) on *h_p_* (5 age groups ×16 trials) confirmed these observations and yielded a significant main effect of age [*F*(4, 49)  = 4.64, *p*<0.001, η^2^ = .53], a significant main effect of trial [*F*(15, 735)  = 6.71, *p*<0.001, η^2^ = .12] and a significant interaction between age group and trial [*F*(60, 735)  = 1.50, *p* = 0.007, η^2^ = .12]. Both significant main effects of trial and interaction exhibited an effect size much lower than age effect size, presumably due to the fact that major part of *h_p_* increase occurred during the five first trials in Age 5–6. Indeed, post-hoc Tukey's HSD tests showed that for Age 5–6, *h_p_* measured for each of the first five trials was significantly different from the following trials. The d value for the difference of *h_p_* between the first and the fifth trials was 0.96. Then, from the sixth trial to the last, post-hoc Tukey's HSD tests also showed a significant difference of *h_p_* between Age 5–6 and each of the other group. For the last trial, the *d* values for the difference of *h_p_* between Age 5–6 and Age 7–8, Age 5–6 and Age 9–10, Age 5–6 and Age 11–12, Age 5–6 and Adults were 1.16, 1.49, 1.24 and 0.93 respectively. Post-hoc Tukey's HSD tests also showed that for Age 7–8, *h_p_* measured for each of the first three trials was significantly different from the following trials while *h_p_* measured of the first trial was significantly different from the following ones for the other age groups.

Repeated measures ANOVA on *SDh_p_* (5 age groups ×16 trials) showed a significant effect of age [*F*(15, 49)  = 26.0, *p*<0.001, η^2^ = .65]. Post-hoc Tukey's HSD tests showed that variability of Adults was significantly lower than that of the other groups, which did not significantly differ between each other on this same variability. The ANOVA also showed a significant decrease of variability with trial repetition [*F*(4, 49)  = 6.81, *p*<0.001, η^2^ = .12], but no significant interaction between age and trial factors.

### Ball-racket impacts

**Figure 3:**
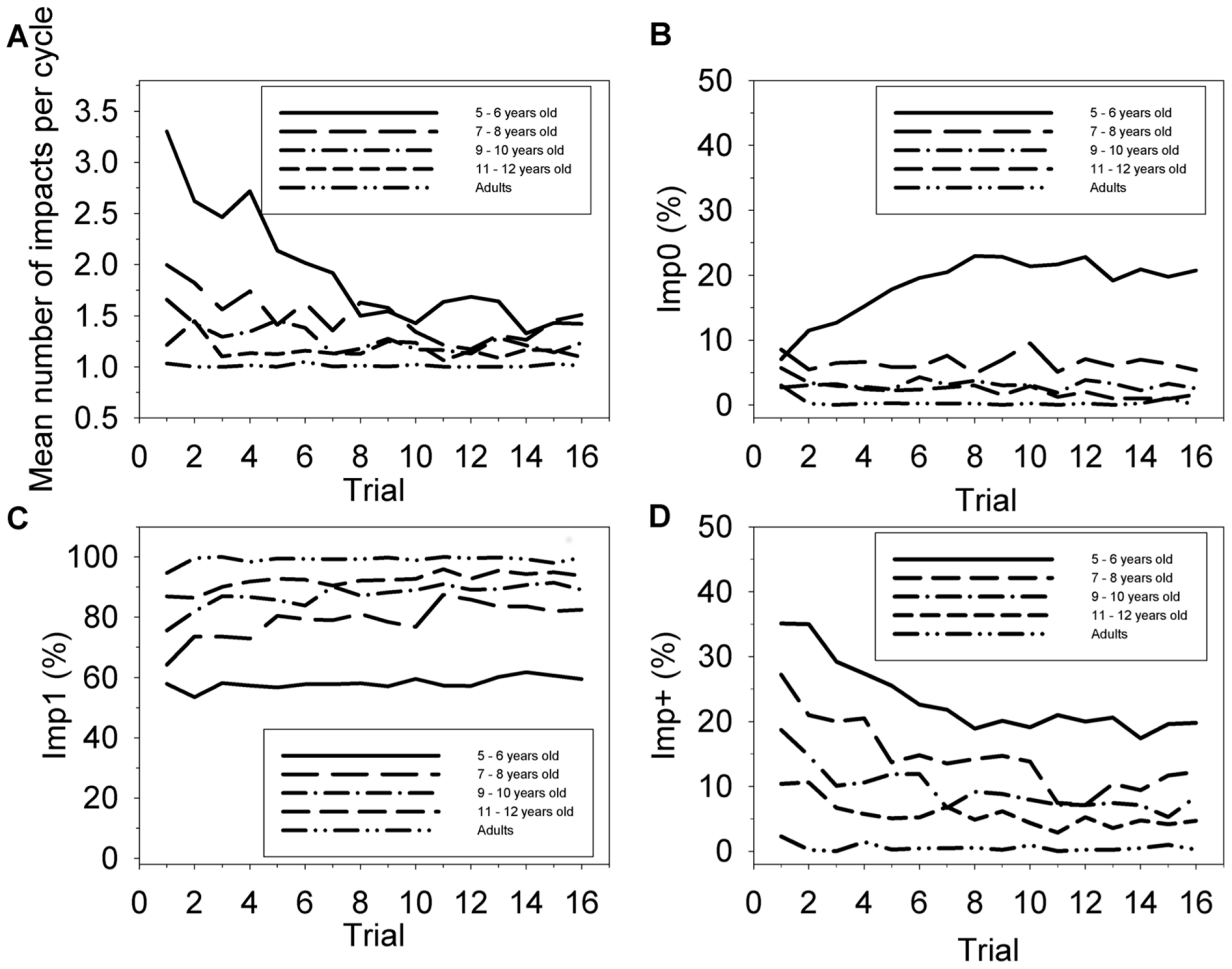
Ball-racket impact. Top-left. Mean number of impacts per racket cycle (*Mean_Imp*) Top-right. Mean percentage of cycles with 1 impact (*Imp1*); Bottom-left. Mean percentage of cycles with 0 impact (*Imp0*); Bottom-right. Mean percentage of cycles with more than 1 impact (*Imp+)*. The variables are plotted as a function of the 16 trials for the 5 age groups.

Participants were expected to perform one racket cycle for each ball cycle, thus 1∶1 bal-racket coordination. As this 1∶1 pattern was not so “trivial” for children, a detailed analysis of ball-racket impacts was required. The mean number of ball impacts per racket cycle in a trial (*Mean_Imp*) yielded large discrepancies between-groups, as shown by Figure 3A. Age 5–6 exhibited a particularly high value of *Mean_Imp* during the very first trials, which corresponds to a tendency to have the ball “stick to the racket”. This result suggests that children from Age 5–6 initially had a hard time bouncing the ball so that it “leaves the racket” and flies (see low values of *h_p_* in previous paragraph). In contrast, older children, from Age 7–8 to Age 11–12, converged more rapidly to the standard value of 1 performed by Adults. These observations were confirmed by a mixed-model ANOVA (age groups × trials) on *Mean_Imp* that yielded a significant main effect of age [*F*(4, 49)  = 16.9, *p*<0.001, η^2^ = .58], of trial [*F*(4, 735)  = 4.23, *p*<0.001, η^2^ = .11] and a significant interaction between age group and trial [*F*(60, 735)  = 2.24, *p*<0.001, η^2^ = .15] (Figure 3A). Post-hoc tests showed that for Age 5–6, each of the first four trials was significantly different from the following trials (the effect size of difference of *Mean_Imp* between the first and the fourth trial was *d* = 0.84).

In order to understand how children managed to decrease *Mean_Imp* with trial repetition, all the cycles of every trial were parted into three categories which were given as percentages: “standard” cycles where only one impact took place (*Imp1* in Figure 3C), cycles with no impacts (*Imp0* in Figure 3B) and cycles with multiple impacts (*Imp+* in Figure 3D). These figures show that the rapid decrease of *Mean_Imp* displayed by Age 5–6, described above, was subtended by a concomitant increase of *Imp0* (from 5% at Trial 1 up to 25% at Trial 8) and decrease of *Imp+* (from 35% to 20%), whilst *Imp1* remained close to 60% throughout the experiment. For all the other groups, *Imp0* remained below 10%, and *Imp+* diminished progressively to converge with repetition towards or below 10%.

Mixed-model ANOVAs (age groups × trials) on *Imp0* and *Imp+* both yielded significant interactions between age group and trial with [*F*(60, 735)  = 2.29, *p*<0.001, η^2^ = .61] and [*F*(60, 735)  = 1.77, *p = *0.007, η^2^ = .11], respectively. For both variables, post-hoc Tukey's HSD tests showed that for Age 5–6 each of the first five trials was significantly different from the following trials. Furthermore, post-hoc Tukey's HSD tests also showed a significant difference for both *Imp0* and *Imp+* between Age 5–6 and Age 7–8 to Adults from the sixth trial to the last. Effect sizes of the difference of *Imp0* between Age 5–6 and other groups were d = 1.21, 2.61, 2.98 and 3.41, respectively, for the last trial. The last trials' effect sizes of the difference of *Imp+* between Age 5–6 and other groups were d = 0.83, 1.95, 3.37 and 6.54 respectively. The ANOVA (age groups × trials) on *Imp1* yielded significant main effects of age group and trial [*F*(4, 49)  = 21.4, *p*<0.001, η^2^ = .63; *F*(15, 735)  = 6.14, *p*<0.001, η^2^ = .11] but no significant interaction.

### Racket oscillation

**Figure 4:**
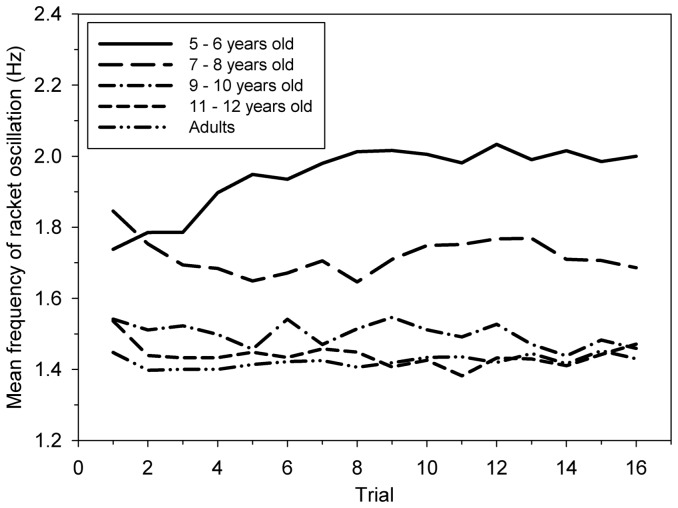
Mean racket frequency (*f_r_*) for the five age groups across trials.

Figure 4 shows that Age 5–6 exhibited an initially unexpected increase in racket frequency *f_r_* with trial repetition, especially between Trial 1 (1.72 Hz) and Trial 5 (1.96 Hz). In contrast, Age 7–8 and Age 8–9 exhibited a decrease, and Age 10–11 and Adults were *fr* almost unchanged (∼1.4 Hz) throughout the experiment. The mixed model ANOVA (age × trial) on *f_r_* yielded a significant main effect of age [*F*(4, 49)  = 15.0, *p*<0.001, η^2^ = .50] and a significant interaction between age group and trial [*F*(60, 735)  = 2.41, *p*<0.001, η^2^ = .14] (Figure 4). Concerning Age 5–6, post-hoc Tukey's HSD tests showed that each of the first five trials was significantly different from the following trials (*d* = 0.83 between the first and the fifth trial). Furthermore, racket frequency of Age 5–6 converged after 5 trials to a limit value of approximately 1.9 Hz, which was significantly different from the reference value of 1.4 Hz produced by adults at the last trial. The adult frequency was not significantly different from theoretical value 1.37 Hz (*t*(9)  = 1.44, *p*>.05).

Variability in racket oscillations was assessed by *SDf_r_*, the standard deviation of racket frequency within a trial. Mixed model ANOVA (5 age groups ×16 trials) on *SDf_r_* yielded a significant main effect of age [*F*(4, 49) = 7,30, *p*<0.001, η^2^ = .51] but no significant effect of trial and no significant interaction. Post-hoc Tukey's HSD test shows that Adult's *SDf_r_* was significantly smaller than *SDf_r_* of all the other groups.

The mixed model ANOVA (age × trial) on the amplitude of the racket oscillation did not yield any significant effects.

**Figure 5:**
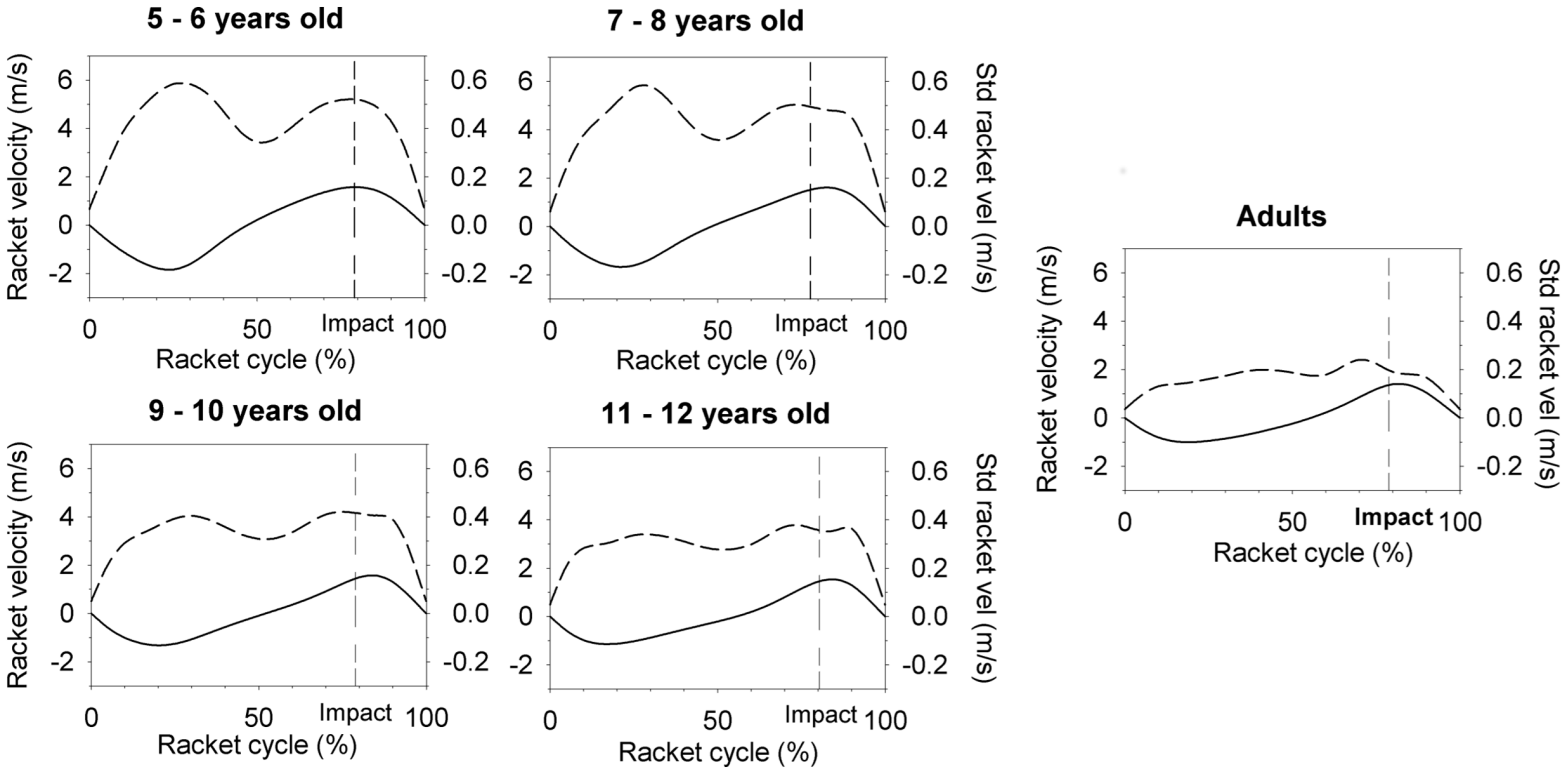
Racket velocity profiles. Mean racket velocity (solid line) and mean within-trial standard deviation (dash line) for the last trial of each age group.

Figure 5 shows the mean velocity profiles of racket cycles for all the groups (for the four last trials), as well as the within-trial variability of the velocity profiles. The amplitudes and “shapes” of the variability profiles exhibit large age-related discrepancies. First, variability is inversely proportional to age, with the highest variability peaks for Age 5–6. More interestingly, the peaks of variability did not occur at the same time in the cycle. For Age 5–6, two peaks of variability occurred at the same time as velocity peaks (whether in the negative or positive range, corresponding to maximum velocity during downswing and upswing, respectively). The second peak of variability also occurred at the same time as impact. Age 7–8 and Age 9–10 also exhibited two peaks of variability, but less pronounced. The mean variability profile of children from Age 11–12 tended to resemble the Adults' profile, which has a plateau shape and where variability is not proportional to velocity.

### Correlation between *T_up_* and *T_r_*

**Figure 6:**
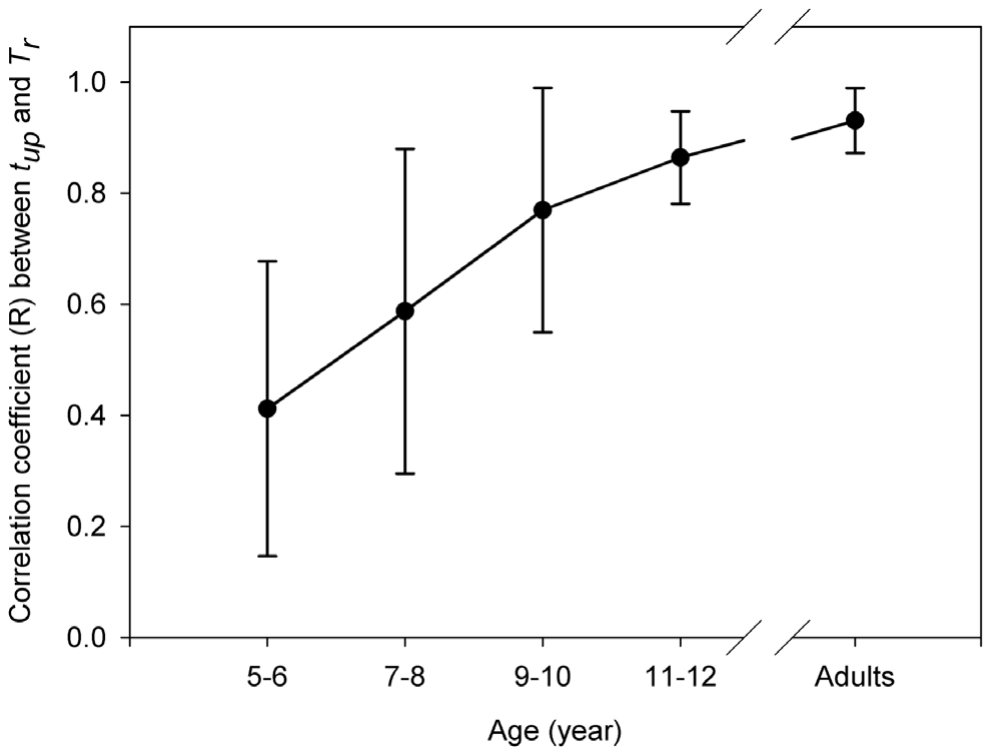
Information-movement coupling. Correlation coefficient (*R_c_*) between duration of the ball upward motion (*T_up_*) of the ball and the period of racket oscillation (*T_r_*) for the 5 age groups. The mean standard deviation *R_c_* for each age group is represented by error bars.

A mixed model ANOVA (5 age groups ×16 trials) on *Rc* (after a Fisher's Z transformation) yielded a significant main effect of age [*F*(4, 49)  = 23.1, *p*<0.001, η^2^ = .62] but no significant effect of trial and no significant interaction. Post-hoc Tukey's HSD tests on age group effect showed that the *Rc* values from Age 5–6 were significantly smaller than *Rc* values of the other groups, characterizing a weaker coupling of racket to ball motion (Figure 6). The effect sizes of the differences of *Rc* for the last trial between Age 5–6 and the other groups were *d* = 0.99, 1.99, 2.95 and 3.57 respectively.

## Discussion

The aim of the present study was to investigate the development of rhythmical visual-motor coordination in children aged from 5 to 12 years. Global improvement in accuracy and stability were expected to mirror a substantial change in both behavioural (qualitative) and performance (quantitative) measures at 7–8 years old. Following the findings of studies on other tasks, we hypothesized that children under age 7 would show restricted visual-motor coordination characterized by a relatively “fixed” frequency racket oscillation and a weak performance. After this age, we expected progressively more accurate performance with increased efficiency of the information-movement coupling involved in the regulation of the task. Finally, we addressed the role of time related visual information involved in the control of the racket period according to the age of the participants.

In general, the dataset supports an overall improvement of performance with age (*h_p_, Mean_Imp, Imp0, Imp+*). The expected changes in both performance and behaviour at the critical age of 7 years old was also observed in the current experiment in several variables (e.g. *h_p_, Mean_Imp, Imp0, Imp+, f_r_, R_c_*). This result is in agreement with previous studies which showed significant changes in both performance and behaviour at the age of 7 years old [14]–[18], [22], [34], [38]–[40]. After the age of 7, the children's ability to perform the task (e.g. *h_p_, Mean_Imp, Imp0, Imp+, R_c_*) kept on increasing with age until the age of 12. It is to be noted that the older children (11–12-year-olds) did not reach the accuracy and the consistency in the task exhibited by adults as reported in previous findings with complex tasks (e.g. [6], [17]]).

Results also showed significant differences across ages in the adaptation to the task. Indeed, up to 6 trials were needed by children under 7 in order to stabilise performance (*h_p_, Mean_Imp, Imp0, Imp+I*) whereas beginning at age 7 the adaptation to the constraints of the task was almost immediate (maximum of two trials required).

Another interesting point deals with the motor behaviour that underlies the children's performance. For Age 5–6, mean ball peak height (*h_p_*) appeared significantly lower than the other age groups (0.45 m versus 0.71 m for young adults) and despite increases of *h_p_* across trials, the youngest children were not able to reach the mean ball height of the other groups. This raises the question how Age 5–6 manage to perform the novel motor task. A very specific performance was observed for Age 5–6 at the very beginning of the task. The youngest children initially had great difficulties bouncing the ball only once per racket cycle, instead exhibiting several ball contacts per racket cycle as if the ball was sticking to the racket. This weak performance was illustrated by both high values of the mean number of ball impacts per racket cycle in a trial (*Mean_Imp)* and the percentage of racket cycles during which more than one impact occurred *(Imp+)*. Moreover, these performance-related observations (*h_p,_ Mean_Imp, Imp+*) reveal the great difficulty displayed by the youngest children to adequately use the kinetic properties of both the racket and the ball to meet the requirements of the task.

After several trials, 5–6 years old children adopted a remarkable ad-hoc behavior in order to meet the requirements of the task. Indeed, increased racket frequency (*fr)* was observed, further stabilizing close to 1.9 Hz, while racket amplitude remained unchanged throughout trials. This 1.9 Hz frequency matches the natural one exhibited by 6-year-old children (1.86 Hz) in a motor tapping task involving synchronisation with a simple tempo sequence [41]. This increase of *fr* resulted in an increase of racket velocity at impact, which can be interpreted as an original motor behaviour which helps to better launch the ball and prevent it from sticking to the racket, as shown by the decrease of *Imp+*. Thus, such specific behaviour could be interpreted as the children's adaptation to their poor ability to regulate racket motion on the basis of visual information, as shown by the small *R_c_* values characterizing the strength of the information-movement coupling. Indeed, throughout the task, *R_c_* values of Age 5–6 remained weak (*R_c_* = 0.37) and significantly smaller than that of the other groups. In other words, the youngest participants were less able than others to take the temporal visual information into account to regulate online racket oscillations. This poor regulation capacity and the high variability in racket motion both yielded very variable ball bounces which in turn might have made it more difficult for children to select proper visual information for the control of racket motion. Furthermore, the low Rc values observed in 5–6 year-old children could reflect of more general limitation of young children to perceive time information. Indeed, Wilkening [42] has shown that younger children are generally less capable of properly integrating two determinants (e.g. judging time as a function of distance and speed). 5–6-year-olds substract speed to distance to estimate travel time, instead of dividing them, whereas 10-year-old children integrate the two determinants properly. This cognitive limitation was also observed in a motor task. Benguigui et al. [43] have shown that across development, children to initially use distance information instead of time information, when asked to judge the moment of arrival of a moving stimulus towards a specified position.

Another explanation of the specific behavior of the younger children could be linked to the absence of haptic feedback when the ball bounces on the virtual racket. Indeed, in adults Sternad et al. showed that if either visual or haptic information are sufficient to sustain the task efficiently, haptic information ensures a more stable behavior than visual information alone [31]. In addition, a series of studies on perceptual-motor coordination reported that children above 8 rely primarily on visual feedback, and children under 8 years old are largely dependent on kinaesthetic feedback [44]–[47]. Therefore, if ball-bouncing was opted in order simplify the task-requirement by limiting perceptual information to vision, the absence of haptic feedback of the impact in the present study could deprive children under the age of 8 of critical information. It could be interesting to test the development of the respective roles of visual and haptic information in the control of the ball bouncing task during childhood.

The improved task control seen in subjects after age 7–8 can be related to the age-related decrease of movement variability. The profiles obtained for the standard deviation of racket velocity (Figure 5) exhibited a decrease of peak variability values as well as a change in the distribution of this variability within the racket cycle. These alterations appear to be related to advancing age. If a classic proportional relationship between standard deviation values and magnitude of velocity with concomitant peaks denoting noise in the sensory-motor system was observed for the youngest participants, a progressive decrease with age of variability could be interpreted as a decrease of noise in the sensory-motor system. Literature has shown that beginning at age of 7–8, children become progressively better at producing faster, more accurate and more consistent motor coordination [17], [48]. The second factor that relates to the improved performance and to adaptability with age is the children's progressive capability to use visual information towards the regulation of movements. Indeed, the racket velocity variability profiles obtained for children Age 7–8 and older progressively converged towards the adults' profile, with a noticeable peak of this variability localised just before the impact. This local peak of variability can be due to the fine racket adjustments using visual information gathered from the ball's trajectory before ball-racket impact which emerges over the course of development These results are consistent with previous literature from Broderick & Newell [49], who observed in a physical ball-bouncing task that variability was not only observed in novice participants but also in experts. Broderick & Newell interpreted the experts' variability in coordination, not as a consequence of sensorimotor noise but as a sensorimotor adjustment ability support. Interpreting the variability observed in the oldest children of our study as adjustment ability is consistent with the increasing values of *Rc* with age, suggesting that the involvement of the information-movement coupling based on visual information in the task regulation improves as children get older. In the same vein, the rapid changes observed in the current experiment at 7 years-old are also consistent with the remarkable improvement of perceptual-motor ability in children [20], [25], [26], [34], [40].

Given the results presented here, the improvement of the ability to perform ball-bouncing across age appears characterized by rapid changes in both behaviour and performance observed at the age of 7. The weak ability to control the racket on the basis of visual information is first proposed in order to explain the poor performance and the remarkable behaviour exhibited by children under 7 years old. In addition, holding a physical racket could be also considered as a factor which potentially affected their performance. Indeed, as pointed by previous literature, using a tool in order to perform a task requires an adaptation of the motor system in order to compensate novel external forces [23], [50]. Surprisingly, our findings related to ability to perform ball-bouncing across age are concordant with developmental trajectory shown by literature related to tool-less tasks such as ball catching or kicking [51]. In opposition, a steady increase in the development of a clubbing or striking pattern in children from 6 to 14 years old has also been shown [51]. For clubbing and striking high velocities and near maximum force is used. Therefore on-line adaptation of the movement is not possible. Virtual ball-bouncing does not require the same force and velocity as in clubbing or striking. This might explain the fact the tennis-table racket used in ball-bouncing does not influence motor coordination in the same way that a tool does in striking or clubbing. It would be interesting to test this hypothesis in a follow-up experiment.

In short, the development of the ability to perform ball bouncing is characterized by changes of both performance and behaviour at the age of 7 years old. Before 7, children's ability to regulate racket movement as a function of the timing of the ball appears to be rather poor. The youngest children coped with this restriction by developing a specific behaviour (by increasing racket frequency) in order to basically meet the task demands. After 7 years old, children become progressively more able to exploit information on ball timing in order to guide racket movement, revealing a qualitatively different information-movement coupling in the task regulation.

## References

[1] Kugler PN, Turvey MT (1987) Information, natural laws, and the self-assembly of rhythmic movements. Hillsdale, NJ: Lawrence Erlbaum.

[2] Getchell N (2006) Age and Task-Related Differences in Timing Stability, Consistency, and Natural Frequency of Children's Rhythmic, Motor Coordination. Dev Psychobiol 48: 675–685. 17111404 10.1002/dev.20186

[3] Thelen E, Fisher DM (1982) Newborn stepping: An explanation for a ‘disappearing’ reflex. Dev Psychol 18: 760–775.

[4] Thelen E (1979) Rhythmical stereotypies in normal human infants. Anim Behav 27: 699–715. 556122 10.1016/0003-3472(79)90006-x

[5] Thelen E (1981) Rhythmical behavior in infancy: An ethological perspective. Dev Psychol 17: 237–257.

[6] Volman MJ, Geuze RH (2000) Temporal stability of rhythmic tapping “on” and “off the beat”: a developmental study. Psychol Res 63: 62–69. 10743387 10.1007/pl00008168

[7] von Hofsten C (1980) Predictive reaching for moving objects by human infants. J Exp Child Psychol 30: 369–382. 7205134 10.1016/0022-0965(80)90043-0

[8] von Hofsten C (1982) Eye-hand coordination in the newborn. Dev Psychol 18: 450–461.

[9] von Hofsten C, Fazel-Zandy S (1984) Development of visually guided hand orientation in reaching. J Exp Child Psychol 38: 208–219. 6491596 10.1016/0022-0965(84)90122-x

[10] Savelsbergh GJ, van der Kamp J (2000) Information in learning to co-ordinate and control movements: is there a need for specificity of practice? Int J Sport Psychol 31: 467–484.

[11] Savelsbergh G, Davids K, van der Kamp J, Bennett SJ, editors (2003) Development of movement coordination in children: applications in the field of ergonomics, health sciences and sport. Routledge.

[12] Fischman MG, Moore JB, Steele KH (1992) Children's one-hand catching as a function of age, gender, and ball location. Res Q Exerc Sport 63: 349–355. 1439158 10.1080/02701367.1992.10608755

[13] Alderson GJ, Sully DJ, Sully HG (1974) An operational analysis of a one-handed catching task using high speed photography. J Mot Behav 6: 217–226. 23961836 10.1080/00222895.1974.10734998

[14] Smoll FL (1975) Variability in development of spatial and temporal elements of rhythmic ability. Percept Mot Skills 40: 140. 10.1080/00222895.1974.10734979 23947410

[15] Smoll FL (1974) Development of spatial and temporal elements of rhythmic ability. J Mot Behav 6: 53–58. 23947410 10.1080/00222895.1974.10734979

[16] Smoll FL (1974) Development of rhythmic ability in response to selected tempos. Percept Mot Skills 39: 767–772.

[17] Ferrel-Chapus C, Hay L, Olivier I, Bard C, Fleury M (2002) Visuomanual coordination in childhood: adaptation to visual distortion. Exp Brain Res 144: 506–517. 12037635 10.1007/s00221-002-1064-2

[18] Lambert J, Bard C (2005) Acquisition of visuomanual skills and improvement of information processing capacities in 6- to 10-year-old children performing a 2D pointing task. Neurosci Lett 377: 1–6. 15722176 10.1016/j.neulet.2004.11.058

[19] Bard C, Hay L (1983) Etude ontogénétique de la coordination visuo-manuelle. Canadian Journal of Psychology/Revue canadienne de psychologie 37: 390–413. 6640446

[20] Olivier I, Ripoll H, Audiffren M (1997) Age differences in using precued information to preprogram interception of a ball. Percept Mot Skills 85: 123–127. 9293568 10.2466/pms.1997.85.1.123

[21] Fitzpatrick P, Schmidt RC, Lockman JJ (1996) Dynamical patterns in the development of clapping. Child Dev 67: 2691–2708.

[22] Getchell N, Whitall J (2003) How do children coordinate simultaneous upper and lower extremity tasks? The development of dual motor task coordination. J Exp Child Psychol 85: 120–140. 12799165 10.1016/s0022-0965(03)00059-6

[23] Konczak JÃ, Jansen-Osmann P, Kalveram KT (2003) Development of force adaptation during childhood. J Mot Behav 35: 41–52. 12724098 10.1080/00222890309602120

[24] Robertson SD (2001) Development of bimanual skill: the search for stable patterns of coordination. J Mot Behav 33: 114–126. 11404208 10.1080/00222890109603144

[25] Debrabant J, Gheysen F, Vingerhoets G, Van Waelvelde H (2012) Age-related differences in predictive response timing in children: Evidence from regularly relative to irregularly paced reaction time performance. Hum Mov Sci 31: 801–810. 22494922 10.1016/j.humov.2011.09.006

[26] Lefebvre C, Reid G (1998) Prediction in ball catching by children with and without a developmental coordination disorder. Adap phys act q 15: 299–315.

[27] Siegler IA, Bardy BG, Warren WH (2010) Passive vs. active control of rhythmic ball bouncing: the role of visual information. J Exp Psychol Hum Percept Perform 36: 729–750. 20515200 10.1037/a0016462

[28] Siegler IA, Bazile C, Warren WH (2013) Mixed control for perception and action: timing and error correction in rhythmic ball-bouncing. Exp Brain Res 226: 603–615. 23515627 10.1007/s00221-013-3475-7

[29] Warren WH (2006) The dynamics of perception and action. Psychol Rev 113: 358–389. 16637765 10.1037/0033-295X.113.2.358

[30] Morice AH, Siegler IA, Bardy BG, Warren WH (2007) Learning new perception-action solutions in virtual ball bouncing. Exp Brain Res 181: 249–265. 17375292 10.1007/s00221-007-0924-1

[31] Sternad D, Duarte M, Katsumata H, Schaal S (2001) Bouncing a ball: tuning into dynamic stability. J Exp Psychol Hum Percept Perform 27: 1163–1184. 11642701 10.1037//0096-1523.27.5.1163

[32] Repp BH, Su YH (2013) Sensorimotor synchronization: A review of recent research (2006–2012). Psychon Bull Rev 20: 403–452. 23397235 10.3758/s13423-012-0371-2

[33] Hove MJ, Iversen JR, Zhang A, Repp BH (2013) Synchronization with competing visual and auditory rhythms: bouncing ball meets metronome. Psychol Res 77: 388–398. 22638726 10.1007/s00426-012-0441-0

[34] Clizbe D, Getchell N (2010) The development of period correction processes in motor coordination: adaptation to temporal perturbation. Motor Control 14: 59–67. 20237403 10.1123/mcj.14.1.59

[35] Morice AH, Siegler IA, Bardy BG (2008) Action-perception patterns in virtual ball bouncing: combating system latency and tracking functional validity. J Neurosci Methods 169: 255–266. 18221787 10.1016/j.jneumeth.2007.11.020

[36] Schaal S, Atkeson CG, Sternad D (1996) One-Handed Juggling: A Dynamical Approach to a Rhythmic Movement Task. J Mot Behav 28: 165–183. 12529218 10.1080/00222895.1996.9941743

[37] Cohen J (1988) Statistical Power Analysis for the Behavioral Sciences (2nd edition). Hillsdale (NJ): Lawrence Erlbaum Associates.

[38] Getchell N (2006) Age and task-related differences in timing stability, consistency, and natural frequency of children's rhythmic, motor coordination. Dev Psychobiol 48: 675–685. 17111404 10.1002/dev.20186

[39] Ringenbach SD, Amazeen PG (2005) How Do Children Control Rate, Amplitude, and Coordination Stability During Bimanual Circle Drawing? Ecological Psychology 17: 1–18.

[40] Getchell N (2007) Developmental aspects of perception-action coupling in multi-limb coordination: rhythmic sensorimotor synchronization. Motor Control 11: 1–15. 17392564

[41] Drake C, Jones MR, Baruch C (2000) The development of rhythmic attending in auditory sequences: Attunement, referent period, focal attending. Cognition 77: 251–288. 11018511 10.1016/s0010-0277(00)00106-2

[42] Wilkening F (1981) Integrating velocity, time, and distance information: A developmental study. Cognit Psychol 13: 231–247. 7226738 10.1016/0010-0285(81)90009-8

[43] Benguigui N, Broderick MP, Baures R, Amorim MA (2008) Motion prediction and the velocity effect in children. British Journal of Developmental Psychology 26: 389–407.

[44] Hay L, Bard C, Fleury M, Teasdale N (1991) Kinematics of aiming in direction and amplitude: A developmental study. Acta Psychol (Amst) 77: 203–215. 1759590 10.1016/0001-6918(91)90035-x

[45] Lantero DA, Ringenbach SD (2007) Developmental differences in the use of visual information during a continuous bimanual coordination task. J Mot Behav 39: 139–155. 17428759 10.3200/JMBR.39.2.139-157

[46] Manyam VJ (1986) A psychophysical measure of visual and kinaesthetic spatial discriminative abilities of adults and children. Perception 15: 313–324. 3797204 10.1068/p150313

[47] Redon C, Hay L, Rigal R, Roll JP (1994) Contribution of the propriomuscular channel to movement coding in children: A study involving the use of vibration-induced kinaesthetic illusion. Hum Mov Sci 13: 95–108.

[48] Schneiberg S, Sveistrup H, McFadyen B, McKinley P, Levin MF (2002) The development of coordination for reach-to-grasp movements in children. Exp Brain Res 146: 142–154. 12195516 10.1007/s00221-002-1156-z

[49] Broderick MP, Newell KM (1999) Coordination Patterns in Ball Bouncing as a Function of Skill. J Mot Behav 31: 165–188. 11177629 10.1080/00222899909600986

[50] Konczak JÃ, vander Velden H, Jaeger L (2009) Learning to play the violin: Motor control by freezing, not freeing degrees of freedom. J Mot Behav 41: 243–252. 19366657 10.3200/JMBR.41.3.243-252

[51] Butterfield SA, Angell RM, Mason CA (2012) Age and sex differences in object control skills by children ages 5 to 14. Percept Mot Skills 114: 261–274. 22582694 10.2466/10.11.25.PMS.114.1.261-274

